# 

**Published:** 1910-11-05

**Authors:** 


**Enlargement of the Spleen.**

As a matter of experience, it is extremely rare to find considerable splenic enlargement in cases of jaundice due to malignant disease in the portal fissure. Backward pressure, as in heart disease, with nutmeg liver does not produce splenic enlargement, but thrombosis of the portal vein, implicating the splenic vein, causes very notable enlargement of the spleen.—*Dr. H. D. Rolleston.*

### APPOINTMENTS AND RESIGNATIONS.

MR. R. H. JOCELYN SWAN, F.R.C.S., formerly assistant surgeon, has been appointed full surgeon, and Mr. H. W. Wilson has been elected an assistant surgeon at the Cancer Hospital; while Dr. Robert Knox, M.R.C.S., has been appointed director of the electrical and radio-therapeutic department.

DR. A. PHILP MITCHELL, resident surgical officer, Chalmers Hospital, Edinburgh, has been awarded the McCunn Scholarship in Surgery (value £100), open to all graduates of medicine of the Universities of Edinburgh, Glasgow, Aberdeen, and St. Andrews of not more than three years' standing.

DR. HAROLD WILTSHIRE, M.B., M.R.C.P., has succeeded Sir Hugh Beevor, F.R.C.P., as assistant physician, at King's College Hospital.

DR. KENNETH ECKENSTEIN, M.R.C.S., has been elected assistant pathologist at the National Hospital for Diseases of the Heart, Soho Square.

DR. A. S. WOODWARK, M.R.C.P., has been appointed assistant curator and demonstrator in the pathological museum at St. Bartholomew's Hospital.

MR. HERBERT CHARLES, M.R.C.S., formerly an assistant, has been made full anæsthetist, and Mr. R. E. Apperley, M.R.C.S., has been appointed a new assistant, at the Middlesex Hospital.

DR. WILLIAM WRIGHT, M.B., D.Sc., F.R.C.S., has been elected Dean of the Medical College, and Mr. Robert Milne, F.R.C.S., has been elected an extra assistant surgeon at the London Hospital.

MR. A. J. WALTON, F.R.C.S., has been appointed assistant surgeon to the Dreadnought Hospital, Greenwich. It is understood, however, that Mr. Walton will still hold the post of surgical registrar to the London Hospital.

The following appointments for November 1 have been made at the General Infirmary, Leeds: Mr. J. L. N. Thoseby, H.P. to Dr. Barrs; Mr. A. W. Hansell, H.S. to Mr. Littlewood; Mr. E. Newhouse, H.S. to Mr. Lawford Knaggs; J. Martin Frobisher, R.M.O. at the Ida Semi-Convalescent Hospital.

### OBITUARY.

THE death is reported at Southborough of Surgeon-Colonel Francis H. Welch, aged seventy-one, who had retired from the Medical Staff. Colonel Welch took his M.R.C.S. in 1860, and became a Fellow in 1871. He was also an L.S.A.

MR. HENRY JOHN BUCK has died at Clapton at the age of seventy-four. He took his M.R.C.S. Eng. in 1859, and in the following year the L.R.C.P. Edin.

THE death is announced at Worthing of Mr. Charles Burnage Penny, aged thirty-four, formerly of Blackheath. He took his diploma in 1903, became a student at the University of London, where he graduated M.B., in 1904.

WE have to announce the death at Hughenden, Coppice Road, Nottingham, of Surgeon-Colonel Chamney Graves Irwin, Medical Staff (retired), aged seventy-nine. In 1853 he took his M.R.C.S., and then proceeded to Dublin, where he graduated M.B. at the University in 1862.

THE death is announced at Northwood of Dr. Robert Edie, of Edinburgh, aged seventy-nine, formerly of Natal.

WE have to record the death of Mr. Hugh Norris, of South Petherton, Somerset, at the age of eighty-nine. Mr. Norris became an L.R.C.S. in 1848, an L.S.A. in 1851, and an L.R.C.P. of Edinburgh eight years later. He was formerly Senior House Surgeon at the Royal Maternity Hospital, Edinburgh, and a member of several scientific or medical societies. Apart from contributions to the medical papers he was author of "South Petherton in the Olden Time."

THE death is announced at Pretoria, South Africa, of Mr. William Barnabas Woodhouse, aged thirty-eight. Mr. Woodhouse took his diploma in 1896, and became L.D.S. in 1897. He studied at the Middlesex Dental Hospital, London, and his English appointments included those of house surgeon to the Royal Dental Hospital, London, and dental surgeon to the Duke of York's School, Chelsea. He was dental surgeon at the Pretoria Hospital, and connected with many scientific and medical societies.

THE following appeal has been issued by the Royal Ear Hospital: "The hospital has, unendowed, for upwards of ninety years been engaged in a quiet way in the alleviation of the subtle and torturing diseases that affect the ear. It is a matter of abiding amazement that work of such profound national and human importance should be left to the enterprise and generosity of private citizens and that those who carry it on should have their work hampered by the accidents and anxieties which are inseparable from anything that is left entirely to private philanthropy." A sum of £5,000 is required, and contributions should be remitted to Sir Ernest Hatch, the hon. treasurer, at 42 and 43 Dean Street, Soho, London, W.

### Gifts and Bequests.

The net personal estate of Miss Florence Nightingale, who died on August 13, is sworn at £35,649, and among various specific and pecuniary legacies she bequeathed the following: To Mother Stanislaus, Rev. Mother of the Hospital Sisters in Great Ormond Street, for her objects, £250; to the Mother Superior of the Devonport Sisters of Mercy, £250; to Miss Crossland, late Home Sister of the Nightingale Training School at St. Thomas's Hospital, an annuity of £50; to Miss Vincent, matron of St. Marylebone Infirmary, an annuity of £30; to Miss Styring, matron of Paddington Infirmary, £100; to Mme. Caroline Werckner, who nursed the French prisoners in the Franco-German War at Breslau, £100; to the managers of the Reading Room at Herbert Hospital, or at Netley or at Aldershot, or at some other place where soldiers may see them, as the executors may decide, the jewels from Queen Victoria and the bracelet from the Sultan, and the other medals and orders, together with an engraving of the ground round Sebastopol; also the bust of her given to her by the soldiers.

---

Mr. Walter Smith, of Brighton, left £50 to the Brighton, Hove, and Preston Dispensary.

The Chelsea Hospital for Women, Fulham Road, has received £100 from Mrs. George Hilditch for the Convalescent Home.

Mr. John Thomas Bunkall, of King's Lynn, Norfolk, and of March, Cambridgeshire, has left £200 to the West Norfolk and Lynn Hospital.

The Treasurers of the Middlesex Hospital have received from the General Mining and Finance Corporation £105 to the late Prince Francis of Teck's Fund, and £5 from Wellington College instead of a wreath in memory of Prince Francis of Teck.

Mrs. Sarah Hancock, of Nottingham Place, London, W. (widow of Mr. James Lyne Hancock, vulcanised india-rubber manufacturer), has left estate of the gross value of £280,379 11s. Her bequests include £1,000 each to the Royal Hospital for Incurables at Putney, the Middlesex Hospital, and the Great Northern Hospital, Holloway.

In response to the appeal of Prince Alexander of Teck, the following donations have been received to the Prince Francis of Teck Memorial Endowment Fund: Mr. J. Bland Sutton, £1,000; Mrs. J. Bland Sutton, £100; the Society of Motor Manufacturers and Traders, £250; the Gramophone Company, £100; Anonymous, £1,000; and Anonymous, an annual subscription of £100.

Mr. John Andrews Humphreys, of Kidderminster, retired grocer, has left £1,000 to the Kidderminster Infirmary, and £500 to the vicar and churchwardens of St. Mary, Kidderminster, upon trust to apply the income in keeping clean and in order his family vaults in the churchyard of that church; but, failing their keeping in order the said vaults, this bequest is to revert to the Kidderminster Infirmary.

Mr. Thomas Vernon Gardner, of Edgbaston, Birmingham, warehouseman, has bequeathed the residue of his property to two nephews and a niece, charged with the payment of the following (among other) legacies on the death of his wife: £100 each to the Birmingham General Hospital; the Queen's Hospital, Birmingham; the Children's Hospital, Broad Street, Birmingham; and the Birmingham and Midland Hospital for Women.

According to the *Jewish Chronicle* Mr. Benjamin Levi has left £1,000 to the Home for Jewish Incurables.

Mr. Frederick Holgate Carwardine, of Lowestoft, has bequeathed £100 to the Royal South London Ophthalmic Hospital.

Mr. Trenly Birch, of Cheltenham, has bequeathed £1,000 each to the General Hospital at Port Elizabeth and the hospital at Grahamstown.

Mr. F. H. Harrison, founder of the Lincoln Malleable Iron and Steel Works, has left £1,000 to Boston Hospital and £200 to the Medical Missions of the C.M.S.

Miss Julia Crosier Sherwood, of Park Mansions, Knightsbridge, has left the residue of her estate equally between St. George's Hospital, London, the Cheltenham General Hospital, and four other charities.

Mr. Thomas Capper, of Reigate, Surrey, and of Messrs. Locke, Lancaster, lead and metal merchants, has left the residue of his property to his son for life, and on his decease £100 to the Redhill and Reigate Cottage Hospital. In a further clause he left, subject to several bequests, the ultimate residue of his property to the children of his said son, and failing issue he bequeathed £1,000 to the London Hospital, and the ultimate residue of his property to King Edward VII.'s Hospital Fund for London. The contingent reversion to London hospitals amounts to about £50,000.

### JOTTINGS.

The receipts of the annual collection of the Hospital Saturday Fund from January to the 15th inst. amounted to £19,028, as compared with £16,477 at the corresponding period of last year, an increase of £2,551.

Princess Louise (Duchess of Argyll) has given her patronage to a bazaar which is being organised by the Ladies' Association of the Victoria Hospital to raise funds to endow a cot in the hospital to the memory of King Edward.

The Duchess of Albany will preside at the final meeting of the Jubilee Committee of the National Hospital for the Paralysed and Epileptic (Albany Memorial), which will be held at the hospital, Queen Square, Bloomsbury, on November 14, at 3 o'clock.

The anniversary of Nottingham General Infirmary was celebrated according to ancient usage for the 128th time last week. Before the service the Governors assembled in the Shire Hall, and, accompanied by the Mayor, Councillor Albert Ball, the Sheriff, Councillor T. Ward, in their robes of office, went in procession to St. Mary's Church.

An appeal is being made for money to start a convalescent home for poor infants, on the ground that there is "no convalescent home in England exclusively for infants, or where the latter can be sent, unless accompanied by their mothers." A beginning has been made at Bury, but it is hoped, if the money comes in, to start again in better quarters at Hunstanton. Subscriptions should be sent to Dr. Stork, M.O.H., Bury St. Edmunds.

We are glad that Sir Riley Lord, Chairman of the Subscriptions Committee of the Royal Victoria Infirmary, Newcastle, has been able to state that the alleged serious loss in the subscription list of the Infirmary, caused by the unfortunate dispute about St. Luke's Chapel, is not borne out by facts. This is greatly to the honour of both Churchmen and Nonconformists; and we hope the public generally will make the facts overwhelmingly clear by providing the increased annual subscriptions needed.

An extension is to be made to King's Cross Hospital at a cost of £2,100. This has been rendered necessary owing to the great increase in the number of admissions, for which the present accommodation is inadequate.

The residents in the University, the town, and the county of Cambridge have unanimously decided that the provision of an extension of Addenbrooke's Hospital, including a new department for out-patients and a children's ward, would be the most suitable memorial to the late King. Promises have been made of £4,000, including £2,000 from Mr. and Mrs. Almeric Paget for the children's ward, £1,000 from Colonel Harding, and £500 from Mr. Charles F. Foster.

Fifty thousand pounds has been subscribed towards the fund for extending the Salford Royal Hospital by all classes of the community, which must be particularly gratifying to Mr. Edward Graham Wood, the chairman of the Extension Committee, who has, with his colleagues, worked unceasingly for the hospital. The borough's memorial to King Edward VII. has been identified with the hospital, and one of the new wings will be called the King Edward Wing.

The Leicester Rugby Football Club has generously decided to devote the gross gate money at its match *v.* Coventry on November 19 to the Leicester Infirmary. The club has given most timely help to the institution during past years, and it is worthy of record that in 1905 £117, and in 1903 £200, was handed over to the infirmary. Mr. Joseph Collier, the club's president, Mr. T. H. Crumbie, the hon. secretary, and Mr. F. St. Clair Pain, the hon. treasurer, are all life governors of the infirmary.

With the sanction of Prince Alexander of Teck (Grand President of the League of Mercy), grants for 1910, amounting in all to £2,190, have been made to extra metropolitan hospitals by the League of Mercy in the following counties: Berkshire, Buckinghamshire, Essex, Hampshire, Hertfordshire, Kent, Middlesex, Oxfordshire, Suffolk, Surrey, and Sussex. The total sum to date handed over to King Edward's Hospital Fund for London is now £135,000, in addition to which £7,661 has been given to extra metropolitan hospitals in counties collecting for the League.

At an extraordinary general meeting of the National Association for the Establishment and Maintenance of Sanatoria for Workers Suffering from Tuberculosis, held at the offices of the Hospital Saturday Fund, 54 Gray's Inn Road, Mr. C. H. Garland, the chairman of the Council, said that despite the extra accommodation provided at Benenden, the original scheme of the association, to erect buildings for two hundred patients, still remained incomplete. The Council appealed to the affiliated societies to subscribe to the building fund. They were gradually reducing the cost of maintenance, and they hoped soon to bring it down to 25s. per bed per week. There was not to-day anything like sufficient accommodation for the large number of consumptives, and the problem could not be properly dealt with until it was taken up by the State. Dr. T. D. Lister, hon. advisory physician to the Council, said that 80 per cent. of the patients discharged from Benenden were found to be still at work up to two years after their discharge.

### TO HOSPITAL SECRETARIES AND OTHERS.

**We invite contributions from any of our readers, and shall be glad to pay, according to length, for items of news for the institutional section (including appointments, resignations, gifts, etc.) which we may publish. All payments for manuscripts used are made as early as possible after the beginning of each quarter.**

### THE MEDICAL SCHOOLS.

THE following are the arrangements for the week at the Medical Graduates' College and Polyclinic, 22 Chenies Street, W.C. : Monday, November 7, at 4 P.M., Dr. Graham Little, Clinique (skin); at 5.15 P.M., Dr. David Ferrier, "Traumatic Neuroses." Tuesday, at 4 P.M., Dr. Porter Parkinson, Clinique (medical); at 5.15 P.M., Mr. F. C. Wallis, "Cancer of the Rectum." Wednesday, at 4 P.M., Mr. James Cantlie, Clinique (surgical); at 5.15 P.M., Mr. F. C. Wallis, "Cancer of the Rectum." Thursday, at 4 P.M., Dr. Walter Carr, Clinique (medical); at 5.15 P.M., Mr. F. C. Wallis, "Cancer of the Rectum." Friday, at 4 P.M., Mr. Ernest Clarke, Clinique (eye).

THE following are the arrangements for the week at the West London Post-graduate College : November 7, pathological demonstration, at 12 noon; November 7 and 10, demonstrations by the Surgical Registrar, at 10 A.M.; November 8, demonstration of minor operations, at 11.30 A.M.; November 9, Dr. Grainger Stewart on "Practical Medicine," at 12.15 P.M.; and November 11, demonstration by the Medical Registrar, at 10 A.M. Lectures at 5 P.M. : November 7, Mr. Rickard Lloyd, on "Anæsthetics"; November 8, Dr. Pritchard, on "Medicine"; November 9 and 10, Mr. Baldwin, on "Practical Surgery"; November 11, Dr. Abraham, on "Cases of Skin Disease."

MR. H. W. WILSON, M.S., F.R.C.S., assistant surgeon to the Victoria Hospital for Children and demonstrator of anatomy at St. Bartholomew's Hospital, has been elected assistant surgeon to the Cancer Hospital, Fulham Road, vice Mr. R. H. J. Swan, F.R.C.S., promoted to the senior staff. Dr. R. Knox, M.D., has been appointed director of the Electrical and Radio-Therapeutic Department at the same hospital.

THE IDEAL WORK OF REFERENCE.

### BURDETT'S HOSPITALS & CHARITIES

*1910 EDITION.*

The Year-Book of Philanthropy and Hospital Annual.

Edited by SIR HENRY BURDETT, K.C.B., K.C.V.O.

A Complete Guide and Directory of the Hospitals, Asylums, and Charitable Institutions throughout the English-speaking World.

Contains Special New Chapters on :—

Voluntary Hospitals in 1909.—Soundness of the Voluntary System.—The need of greater zeal on the part of those who accept office.—State-aided Hospitals in the U.S.A. and Canada.—The Appropriation of Public Funds for the partial Support of Voluntary Hospitals in U.S.A. and Canada.—The Need of an Apostle and of greater interest on the part of the Clergy, &c., &c.

*TWENTY-FIRST YEAR OF PUBLICATION.*

**Over 1,000 pages. Price 10/6 net; Post Free, 10/11.**

ORDER your copy of the Medical Whitaker **NOW.**

Publishers:
THE SCIENTIFIC PRESS, LIMITED, 28 & 29 Southampton Street, Strand, London, W.C.

